# Supplementary material for: Behavioral and neuronal underpinnings of safety in numbers in fruit flies
Source: Nat Commun. 2020 Aug 21;11:4182. doi: 10.1038/s41467-020-17856-4 (PMC7442810; doi:10.1038/s41467-020-17856-4)
Supplement: Supplementary file 1 — Supplementary Information [file 41467_2020_17856_MOESM1_ESM.pdf]

Supplementary Information

Behavioral and neuronal underpinnings of safety in numbers in fruit flies

Ferreira, CH and Moita, MA

Correspondence:

clara.ferreira@neuro.fchampalimaud.org

marta.moita@neuro.fchampalimaud.org

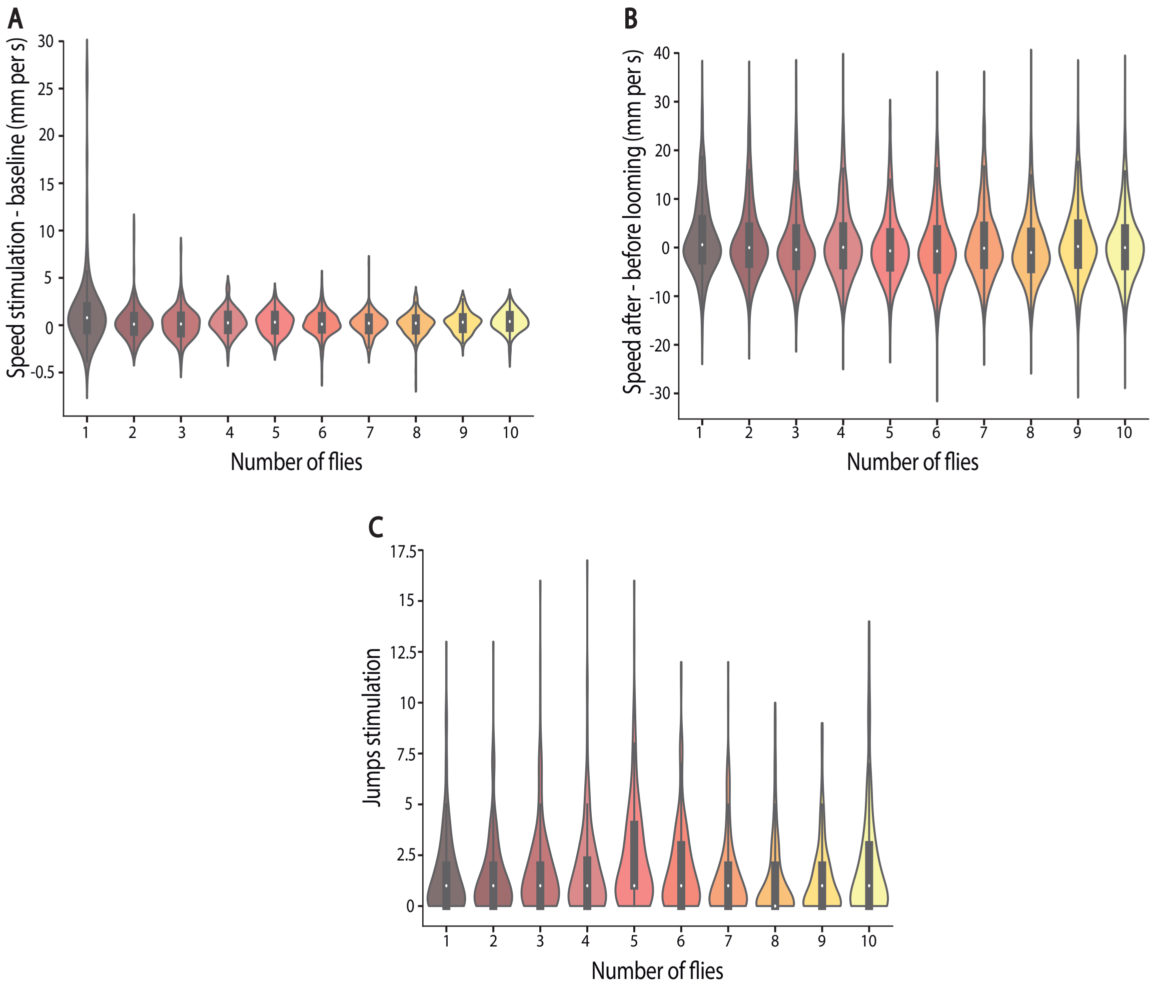

Supplementary Figure 1. Group effect on running and jumps. A–C) Violin plots representing the probability density of individual fly data bound to the range of possible values, with boxplots elements: center line, median; box limits, upper (75) and lower (25) quartiles; whiskers, 1.5x interquartile range). A) Difference in speed between the baseline and stimulation. B) Speed differences 1 s around looming. C) Number of jumps during the stimulation.

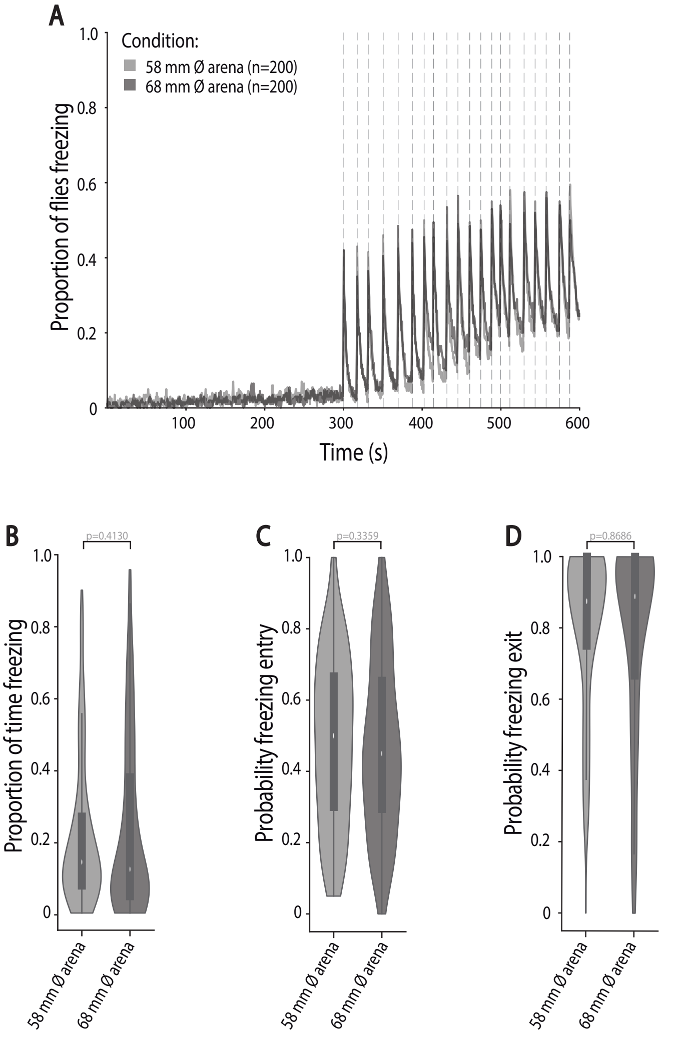

Supplementary Figure 2. Density effect on freezing behaviour in groups of 5 flies. A) Proportion of flies freezing throughout the experiment, for groups of 5 flies in 58 mm diameter (Ø) arenas or in 68 mm diameter arenas (standard arena). B–D) Violin plots representing the probability density of individual fly data bound to the range of possible values, with boxplots elements: center line, median; box limits, upper (75) and lower (25) quartiles; whiskers, 1.5x interquartile range). B) Proportion of time spent freezing throughout the experiment. C) Probability of freezing entry after looming presentation. D) Probability of freezing exit before the following looming stimulus. *P*-values result from two-tailed Mann-Whitney test.

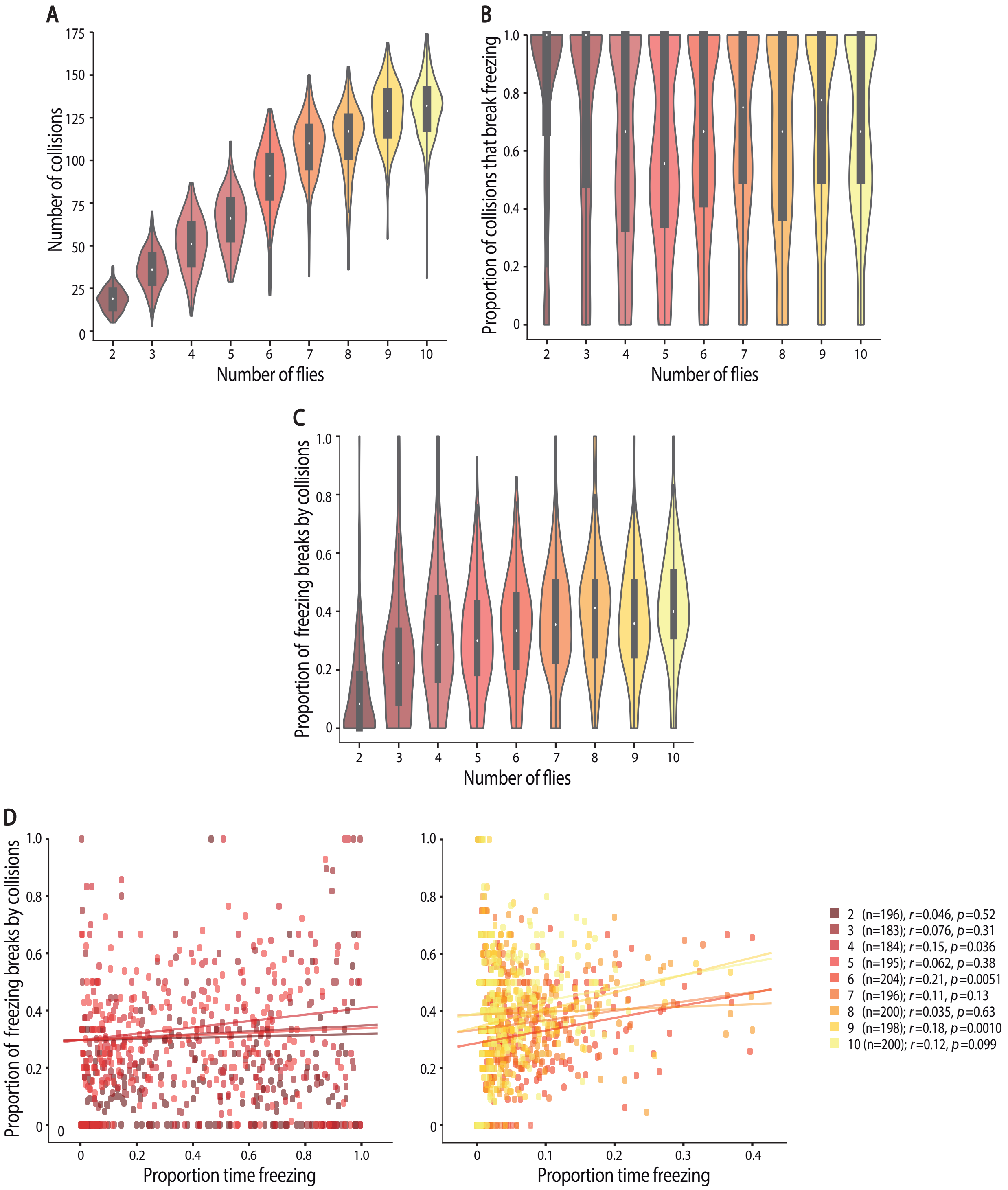

Supplementary Figure 3. The role of collisions on the group effect on freezing responses. A–C) Violin plots representing the probability density of individual fly data bound to the range of possible values, with boxplots elements: center line, median; box limits, upper (75) and lower (25) quartiles; whiskers, 1.5x interquartile range). A) Number of collisions throughout the experiment. Statistical comparisons between conditions presented in Supplementary Table 4. B) Proportion of collisions that break freezing. Statistical comparisons between conditions presented in Supplementary Table 5. C) Proportion of freezing breaks by collision. Statistical comparisons between conditions presented in Supplementary Table 6. D) Correlation between the proportion of freezing breaks by collision and the proportion of time freezing for groups of 2 to 5 (left) and groups of 6 to 10 individuals (right).

The number of collisions an individual experiences increased with the increase in group size and collisions were very effective at breaking freezing. However, although the proportion of freezing breaks due to collisions increased with group size, even in groups of 10 individuals, where collisions only led to 40% IQR 31.58-53.725% of freezing breaks. Furthermore, the fact that the correlation between freezing breaks by collision and proportion of time freezing, were inexistent or very weak, further shows that collisions only partly contribute to the group effect on freezing.

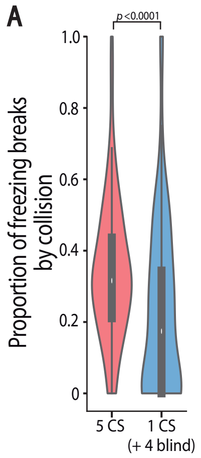

Supplementary Figure 4. Effect of manipulating the motion signal with blind flies on the proportion of freezing breaks by collision. A) Violin plot representing the probability density of individual fly data bound to the range of possible values, with boxplots elements: center line, median; box limits, upper (75) and lower (25) quartiles; whiskers, 1.5x interquartile range). *P*-value results from two-tailed Mann-Whitney test. Since the number of freezing breaks by collision decreased in groups with blind flies, the decrease in freezing observed in these groups cannot be accounted for by contacts between flies.

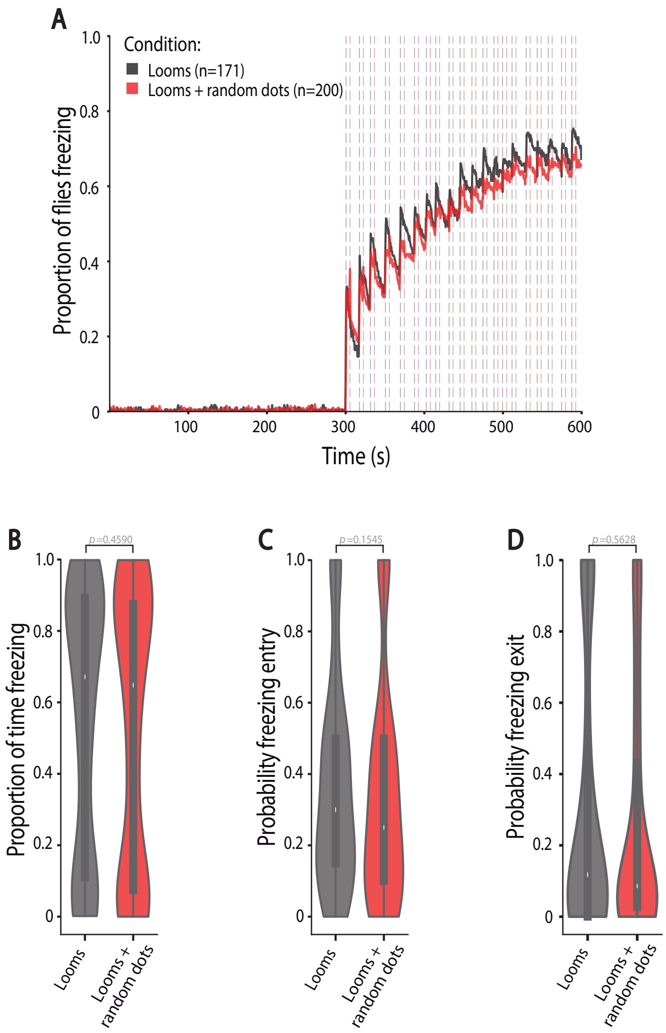

Supplementary Figure 5. The effect of visual disruption after looming. A) Proportion of flies freezing throughout the experiment, for individuals exposed to loomings alone or looms followed by random dots. B–D) Violin plots representing the probability density of individual fly data bound to the range of possible values, with boxplots elements: center line, median; box limits, upper (75) and lower (25) quartiles; whiskers, 1.5x interquartile range). B) Proportion of time spent freezing throughout the experiment. C) Probability of freezing entry after looming presentation. D) Probability of freezing exit before the following looming stimulus. *P*-values result two-tailed Mann-Whitney test.

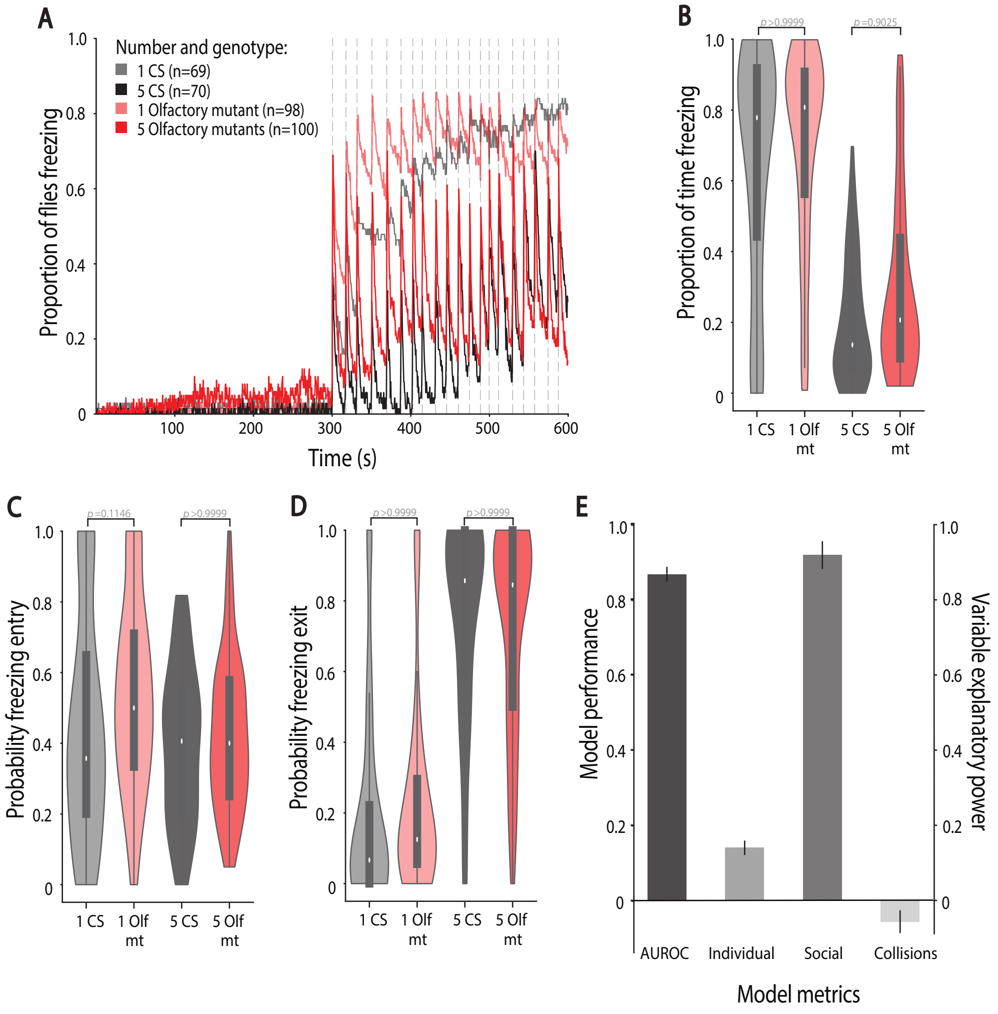

Supplementary Figure 6. The role of other sensory modalities on social regulation of freezing responses in groups. A) Proportion of flies freezing throughout the experiment for flies tested individually and in groups of 5 wild-type flies (CS) and near-anosmic olfactory mutants (mt). B–D) Violin plots representing the probability density of individual fly data bound to the range of possible values, with boxplots elements: center line, median; box limits, upper (75) and lower (25) quartiles; whiskers, 1.5x interquartile range). B) Proportion of time spent freezing throughout the experiment. C) Probability of freezing entry in the 500 ms bin following looming presentation. D) Probability of freezing exit determined in the 500 ms bin before the following looming stimulus. *P*-values result from Kruskal-Wallis statistical analysis followed by Dunn's multiple comparisons test. E) Adding collisions to the logistic regression model presented in Figure 3, does not improve the model's predictive capability; the fact that contacts do not seem to be important for the group response argues against a role for gustation in this process. Mechanosensation may still play a role but see discussion section.

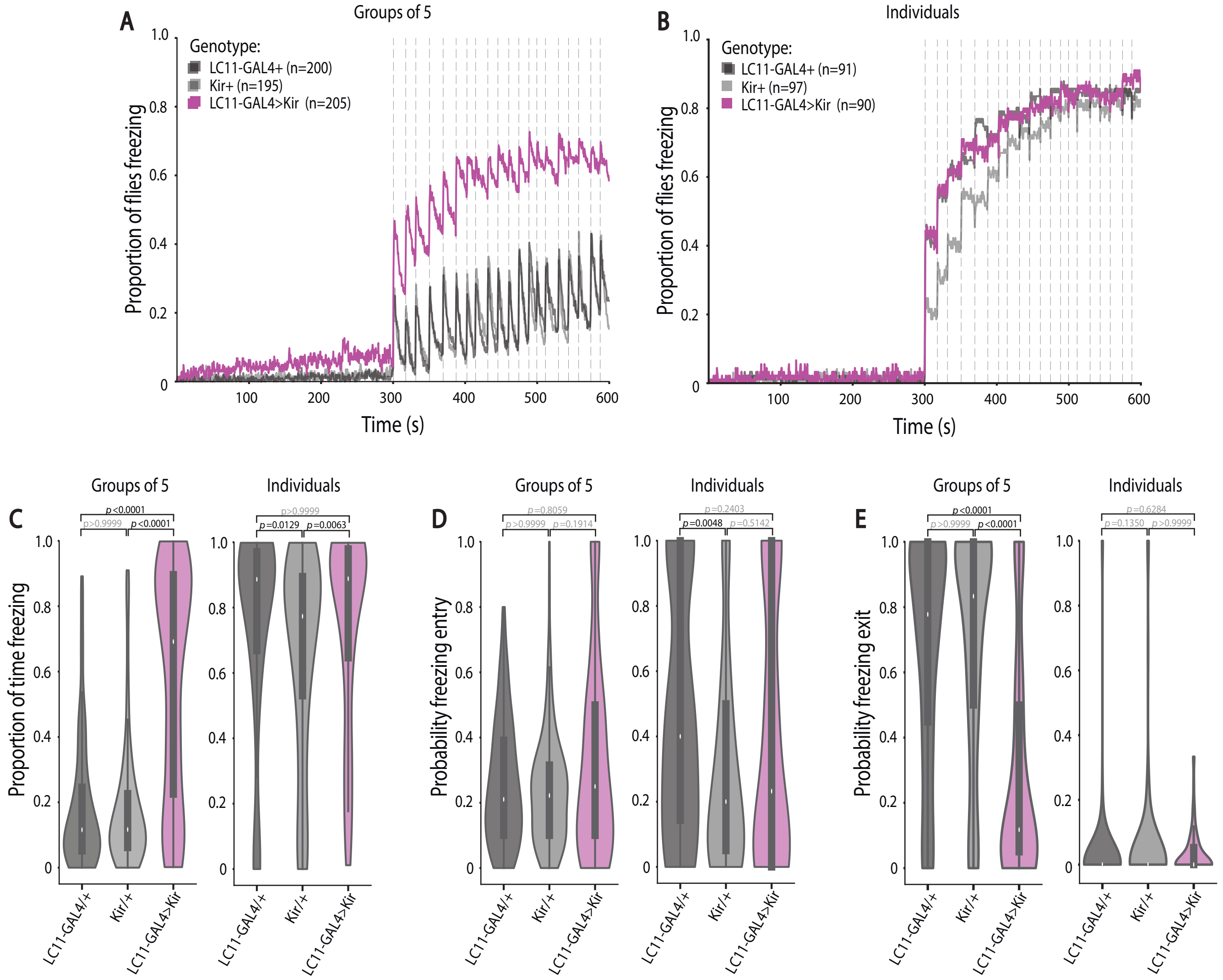

Supplementary Figure 7. Manipulating lobula columnar neurons 11 (LC11) only affects group behaviour. A–B) Proportion of flies freezing throughout the experiment, for A) groups of 5 *LC11-GAL4>Kir2.1* and B) individually tested *LC11-GAL4>Kir2.1* flies depicted in purple and parental controls (grey). C–E) Violin plots representing the probability density of individual fly data bound to the range of possible values, with boxplots elements: center line, median; box limits, upper (75) and lower (25) quartiles; whiskers, 1.5x interquartile range). C) Proportion of time spent freezing throughout the experiment. D) Probability of freezing entry after looming presentation. E) Probability of freezing exit before the following looming stimulus. *P*-values result from Kruskal-Wallis statistical analysis followed by Dunn's multiple comparisons test.

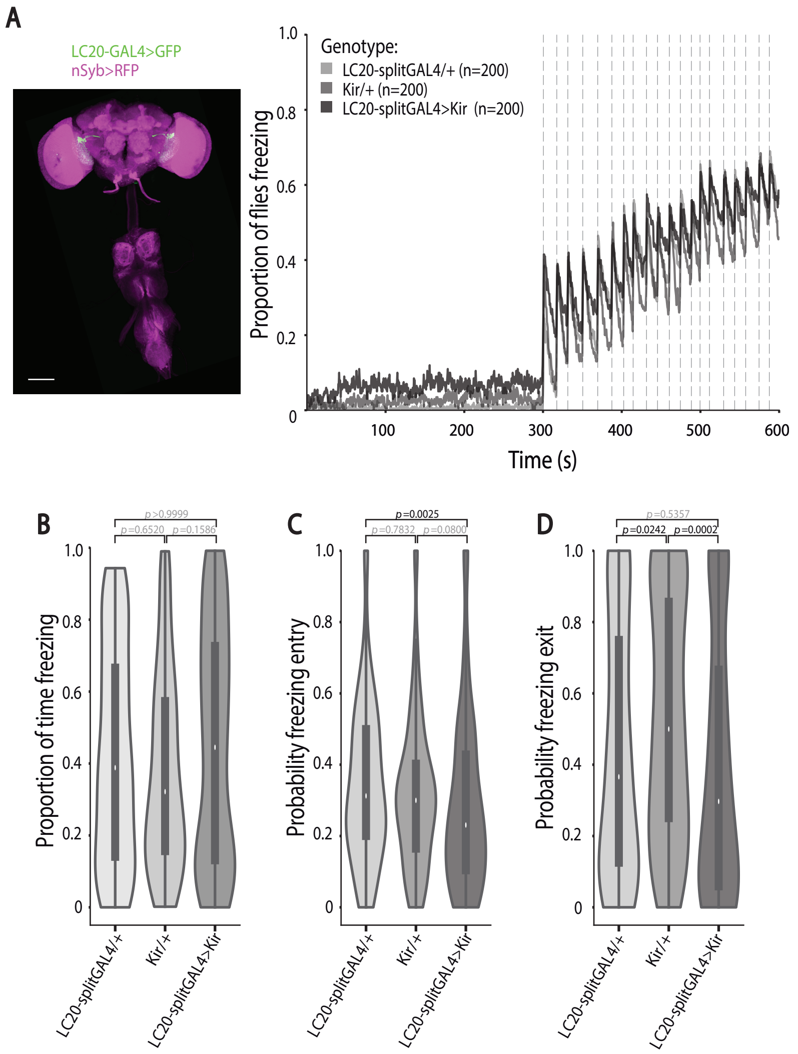

Supplementary Figure 8. Manipulating lobula columnar neurons 20 (LC20). A) Anatomy (scale bar, 100 µm) and proportion of flies freezing throughout the experiment in groups of 5, for *LC20-splitGAL4>Kir2.1* in dark grey, compared to parental controls (light grey). B–D) Violin plots representing the probability density of individual fly data bound to the range of possible values, with boxplots elements: center line, median; box limits, upper (75) and lower (25) quartiles; whiskers, 1.5x interquartile range). B) Proportion of time spent freezing throughout the experiment. C) Probability of freezing entry after looming presentation. D) Probability of freezing exit before the following looming stimulus. *P*-values result from Kruskal-Wallis statistical analysis followed by Dunn's multiple comparisons test.

Supplementary Table 1. Statistical comparisons of the time each fly spent freezing when tested individually and in groups of up to 10 individuals (*p*-values for Kruskall-Wallis followed by Dunn's multiple comparisons test).

	2	3	4	5	6	7	8	9	10
1	0.0272	0.0229	0.0009	0.0001	<0.0001	<0.0001	<0.0001	<0.0001	<0.0001
2		>0.9999	>0.9999	>0.9999	<0.0001	<0.0001	<0.0001	<0.0001	<0.0001
3			>0.9999	>0.9999	<0.0001	<0.0001	<0.0001	<0.0001	<0.0001
4				>0.9999	<0.0001	<0.0001	<0.0001	<0.0001	<0.0001
5					<0.0001	<0.0001	<0.0001	<0.0001	<0.0001
6						0.1189	0.5697	<0.0001	0.0004
7							>0.9999	>0.9999	>0.9999
8								0.5132	>0.9999
9									>0.9999

Supplementary Table 2. Statistical comparisons of the probability each fly started freezing after a looming stimulus when tested individually and in groups of up to 10 individuals (*p*-values for Kruskall-Wallis followed by Dunn's multiple comparisons test).

	2	3	4	5	6	7	8	9	10
1	>0.9999	>0.9999	>0.9999	>0.9999	>0.9999	<0.0001	0.0007	<0.0001	<0.0001
2		>0.9999	>0.9999	0.5177	>0.9999	<0.0001	0.0114	<0.0001	<0.0001
3			>0.9999	>0.9999	0.1128	<0.0001	<0.0001	<0.0001	<0.0001
4				>0.9999	>0.9999	<0.0001	0.0011	<0.0001	<0.0001
5					0.0029	<0.0001	<0.0001	<0.0001	<0.0001
6						0.0113	>0.9999	0.0003	0.0009
7							>0.9999	>0.9999	>0.9999
8								>0.9999	>0.9999
9									>0.9999

Supplementary Table 3. Statistical comparisons of probability each fly stopped freezing before the following looming stimulus when tested individually and in groups of up to 10 individuals (*p*-values for Kruskall-Wallis followed by Dunn's multiple comparisons test).

	2	3	4	5	6	7	8	9	10
1	<0.0001	<0.0001	<0.0001	<0.0001	<0.0001	<0.0001	<0.0001	<0.0001	<0.0001
2		>0.9999	>0.9999	0.0395	<0.0001	<0.0001	<0.0001	<0.0001	<0.0001
3			>0.9999	0.3299	<0.0001	<0.0001	<0.0001	<0.0001	<0.0001
4				>0.9999	<0.0001	<0.0001	<0.0001	<0.0001	<0.0001
5					<0.0001	<0.0001	<0.0001	<0.0001	<0.0001
6						>0.9999	>0.9999	>0.9999	>0.9999
7							>0.9999	>0.9999	>0.9999
8								>0.9999	>0.9999
9									>0.9999

Supplementary Table 4. Statistical comparisons of the number of collisions each fly experienced throughout the experiment when tested in groups of 2 to 10 individuals (*p*-values for Kruskall-Wallis followed by Dunn's multiple comparisons test).

	3	4	5	6	7	8	9	10
2	0.0014	<0.0001	<0.0001	<0.0001	<0.0001	<0.0001	<0.0001	<0.0001
3		0.0664	<0.0001	<0.0001	<0.0001	<0.0001	<0.0001	<0.0001
4			0.1555	<0.0001	<0.0001	<0.0001	<0.0001	<0.0001
5				<0.0001	<0.0001	<0.0001	<0.0001	<0.0001
6					0.0001	<0.0001	<0.0001	<0.0001
7						>0.9999	<0.0001	<0.0001
8							0.0013	<0.0001
9								>0.9999

Supplementary Table 5. Statistical comparisons of the proportion of collisions that led to freezing exit per fly when tested in groups of 2 to 10 individuals (*p*-values for Kruskall-Wallis followed by Dunn's multiple comparisons test).

	3	4	5	6	7	8	9	10
2	0.1090	<0.0001	<0.0001	<0.0001	0.0013	<0.0001	0.0120	0.0002
3		0.3963	0.0126	>0.9999	>0.9999	0.2947	>0.9999	>0.9999
4			>0.9999	>0.9999	>0.9999	>0.9999	>0.9999	>0.9999
5				>0.9999	0.4059	>0.9999	0.0732	>0.9999
6					>0.9999	>0.9999	>0.9999	>0.9999
7						>0.9999	>0.9999	>0.9999
8							0.9607	>0.9999
9								>0.9999

Supplementary Table 6. Statistical comparisons of the proportion of freezing breaks by collision per fly when tested in groups of 2 to 10 individuals (*p*-values for Kruskall-Wallis followed by Dunn's multiple comparisons test).

	3	4	5	6	7	8	9	10
2	<0.0001	<0.0001	<0.0001	<0.0001	<0.0001	<0.0001	<0.0001	<0.0001
3		0.0636	0.0258	<0.0001	<0.0001	<0.0001	<0.0001	<0.0001
4			>0.9999	>0.9999	0.3180	0.0002	0.0275	<0.0001
5				>0.9999	0.5244	0.0003	0.0494	<0.0001
6					>0.9999	0.1155	>0.9999	0.0065
7						>0.9999	>0.9999	0.1938
8							>0.9999	>0.9999
9								>0.9999
